# The opioid mortality epidemic in North America: do we understand the supply side dynamics of this unprecedented crisis?

**DOI:** 10.1186/s13011-020-0256-8

**Published:** 2020-02-17

**Authors:** Benedikt Fischer, Michelle Pang, Wayne Jones

**Affiliations:** 1 Schools of Population Health and Pharmacy, Faculty of Medical and Health Sciences, University of Auckland, Auckland, New Zealand; 2 Department of Psychiatry, University of Toronto, Toronto, Ontario Canada; 3 Department of Psychiatry, Federal University of São Paulo (UNIFESP), São Paulo, Brazil; 4 Centre for Applied Research in Mental Health and Addiction (CARMHA), Faculty of Health Sciences, Simon Fraser University, Vancouver, British Columbia Canada

**Keywords:** Illicit opioids, Prescription opioids, Supply, Demand, Control, Mortality, Policy, Public health, North America

## Abstract

While there has been extensive attention to the ‘demand side’ – or use and adverse consequences, including mortality – of the ‘opioid crisis’ presently unfolding across North America, few considerations have focused on the supply side. This paper examines the supply side dynamics of this unprecedented public health phenomenon. We provide evidence for several interrelated supply-side elements that have contributed to the present public health crisis. We observe that initially, persistently high levels of prescription opioid availability and use exposed large proportions of the North American population to opioids, resulting in correspondingly high levels of medical and non-medical use (e.g., involving diversion). While various intervention measures to control prescription opioid availability and use have been implemented in recent years, leading to eventual reductions in opioid dispensing levels, these occurred late in the crisis’s evolution. Moreover, these supply reductions have not been met by corresponding reductions in opioid use or demand levels. These growing discrepancies between opioid demand and prescription-based sources have left major gaps in opioid supplies. In response to such supply gaps, highly potent and toxic illicit opioid products have rapidly proliferated across North America, and become a core driver of the dramatic spikes in opioid overdose fatality levels in recent years. These supply-related interrelations are corroborated by a corresponding increase in illicit opioid-related fatalities, which arose just as medical opioid supplies began to decrease in many jurisdictions. Improved analyses and understanding of the supply-side dynamics of the opioid crisis are urgently needed in order to inform future intervention and policy development. Meanwhile, the high mortality toll related to illicit, highly toxic opioid exposure requires sustained solutions, including supply-oriented measures (e.g., safer opioid distribution for at–risk users) towards improved public health protection.

## Background

North America has been experiencing an unprecedented public health crisis involving the non-medical use of opioid drugs, and consequentially, a massive toll of accidental opioid-related mortality (i.e., overdose). In 2018, there were 47,590 opioid-related deaths in the United States and a corresponding 4,614 opioid-related deaths in Canada; a threefold increase from a mere decade ago, [1, 2]. In both countries, opioid-related mortality has led to discernable reductions in life expectancy across the general population [3, 4]. Naturally, the lion’s share of scientific attention has been devoted to examining risk factors or outcomes for morbidity and mortality as well as assessing intervention measures in reducing deaths among opioid users (i.e., the ‘demand side’) [5–8]. Much less attention, however, has been devoted to examining aspects of the ‘supply side’ of this unprecedented public health crisis.

## Main text

The emergence of highly potent and toxic synthetic opioid products (e.g., fentanyl and analogues) has been identified as the distinct contributing factor to recent spikes in opioid-related mortality in the United States (U.S.) and Canada [9, 10]. These illicitly produced and distributed opioid products have appeared and proliferated in North America only during recent (i.e., past five) years, with many distributed as counterfeit prescription (including prescription opioid) drugs or mixed in with other drug products (e.g., heroin or cocaine) which render ready detection - either by consumers or law enforcement - highly difficult [11, 12]. To illustrate: in Canada, the steepest increases in recent opioid-related deaths have occurred in the Western provinces, where over three quarters of opioid-related deaths in British Columbia and Alberta have involved fentanyl or fentanyl-analogues [13, 14]. In the U.S., mortality related to synthetic opioids, despite stark regional differences (e.g., increases in mortality observed mainly in the North-Eastern states), has risen by approximately 300% between 2013 and 2017, far outpacing deaths related to other opioids in the years following 2015 [15]. Synthetic opioids, primarily in the form of fentanyl, have also been detected in a growing number of non-opioid-related overdoses, including roughly half of all cocaine-related fatal overdoses in the U.S. in 2017 [16]. In line with these dynamics, the present opioid mortality crisis has been widely characterized as a ‘fentanyl’ epidemic, consequently implicating synthetic opioid drugs as the primary culprit of the unprecedented death toll, although their predominant role and impact has been regionally inconsistent and heterogeneous across North America [10, 17].

An important challenge therefore remains in adequately understanding the ‘supply side’ dynamics of the present opioid death crisis. Illicit drug markets and related dynamics are complex phenomena involving multitudinal factors, many of which are difficult to dissect and understand yet essential for evidence-based analysis and interventions [18]. One documented instance is the Australian ‘heroin drought’, where a sudden shortage in the nation’s heroin supply resulted in subsequent shifts to other non-opioid illicit drug use and related harms during the mid-1990s [19, 20]. It has never been fully clear why the relatively robust Australian heroin market was unable to adjust to the disruption of one supply channel [19]. Similarly, the supply side dynamics of the North American opioid crisis - even rudimentarily – do not seem to be well-examined or -understood, and few efforts have sought to assess why and how North American opioid markets were suddenly flooded with a growing supply of synthetic opioids, resulting in substantial social and health-related harms. Has the influx of synthetic opioids in the U.S. and Canada been an independent event - a kind of ‘supply shock’ event driven predominantly by factors external to the North American markets? Or rather, were the observed shifts in drug supply a response to existing domestic demand-side forces? Select examinations have sought to explore this question, including a ‘three-wave’ characterization of recent opioid supply, yet findings seem to primarily emphasize evolutionary developments in international illicit opioid production and distribution chains as the main drivers behind the opioid crisis unfolding in North America [21, 22].

Substantial evidence supports the former perspective, namely that the recent 'wave' of toxic illicit opioid supply has emerged as an interrelated consequence within pre-existing opioid demand and supply dynamics. The broader context of the phenomenon of increasing opioid-related mortality in North America originally evolved in the early 2000s, centrally driven by substantial increases in population-level dispensing of prescription (medical) opioids, consequent increases in non-medical use, and related morbidity and mortality [23]. Specifically, consumption rates of prescription opioids (POs; in defined daily doses [DDD] per population/day) rose by several magnitudes in North America between 2001 and 2013, rendering the U.S. and Canada the two countries with the highest levels of opioid consumption globally [24]. During peak years of medical PO availability, between 2010 and 2012, as many as one-in-five of general population adults reported annual PO use, while approximately 5% were involved in annual ‘non-medical’ use [23]. Moreover, the prevalence of PO use disorder in the U.S. general population increased by 50% between 2003 and 2013, to about 1% of the general population [25]. Taken together, these data illustrate the realities of the persistently flush PO environments in North America, fuelled largely by extensive medical system-based dispensing of opioids. Aside from vastly broadening the population base of opioid users, these developments further led to paradigmatic shifts in the supply path for non-medical opioid use during the early 2000s. While local heroin markets, and use continued to exist in select locations, heroin was largely replaced with medically sourced or diverted POs in the early 2000s even among marginalized (e.g., street-involved) users, as documented by several North American field studies [26, 27]. This shift centrally included slow-release oxycodone (e.g., Oxycontin) but also other commonly available medical PO products, such as fentanyl, hydromorphone, and hydrocodone formulations.

These distinct increases in medically sourced or diverted opioid product supplies in North America were gradually reflected in patterns of drug-related mortality. Both the U.S. and Canada began experiencing substantial increases in opioid-related deaths due to PO-related fatalities between 2002 and 2012 [23, 28, 29], with several epidemiological examinations demonstrating strong correlations between population-levels of dispensing and mortality related to individual PO formulations during this period [30–32]. Within merely a decade, large portions of the North American population across all socio-economic strata had become habituated into medical and non-medical opioid use, and as a dire consequence, both Canada and the U.S. experienced sharply increasing levels of opioid-related morbidity (e.g., emergency room admissions, opioid dependence diagnoses) and overdose-related deaths unseen elsewhere in the world [23, 33, 34].

Between 2010 and 2012, North American intervention systems began to more actively respond to the emerging PO-related crisis, enacting and/or reinforcing a multitude of interventions aimed at constraining the excessive levels of PO-related harms [5, 35]. Such measures included, for example, new and improved prescription monitoring systems to detect over-use and -prescribing, the introduction of ‘abuse deterrent’ opioid formulations, restrictions (e.g., formulary-based) on opioid availability, revised (more restrictive) opioid prescribing guidelines and enforcement measures to target sources of overprescribing and diversion (e.g., ‘pill mills’) among others [6, 36–38]. While it is impossible to attribute precise effects to individual measures, these actions have jointly led medical PO dispensing levels to plateau or decline across select North American jurisdictions post-2012 [39, 40]. Concretely, based on several data sources, population levels of PO-dispensing declined by about 20% in Canada [40], and about half of all provinces – notably those with subsequently high numbers of illicit opioid-related mortality – experienced substantially greater declines post-2010 [40, 41]. Correspondingly, PO dispensing in the US (e.g., as measured in morphine equivalents) declined by similar proportions, between 2010 and 2016, although with substantially heterogeneous inter-state patterns [42]. While globally, North America remained the regional leader in PO consumption, medical opioid availability and dispensing became increasingly more restrained and strictly monitored, naturally translating into decreased availability and diversion potential for non medical opioid use. This left multiple but large sub-groups of individuals with demand for opioids with shrinking supplies of non-medical access to opioids, including those with main motives to control pain as well as those with of non-medical use. A concurrent indicator of declining supplies has been the price-levels of diverted POs, which rose substantially in many North American settings [43, 44]. Importantly, however, the population-level demand for opioids, and levels of non-medical or problematic opioid use, did not correspondingly decrease, but rather remained elevated or continued to climb [25, 45, 46]. Availability of and engagement in treatment for opioid disorders have seen substantial expansions in North America over the past decade, yet these observations have been limited to minority sub-populations actively seeking and engaging in treatment and rehabilitation, leaving sizeable non-medical user populations exposed to the overall contractions in opioid supply [46, 47].

**Fig. 1:**
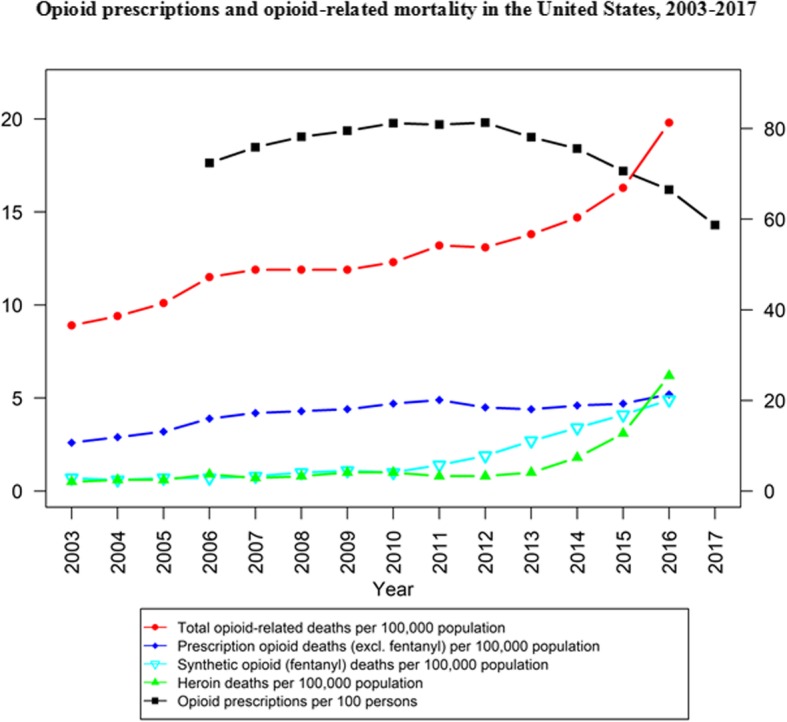
Opioid prescriptions and opioid-related mortality in the United States, 2003–2017 [66, 67]

**Fig. 2:**
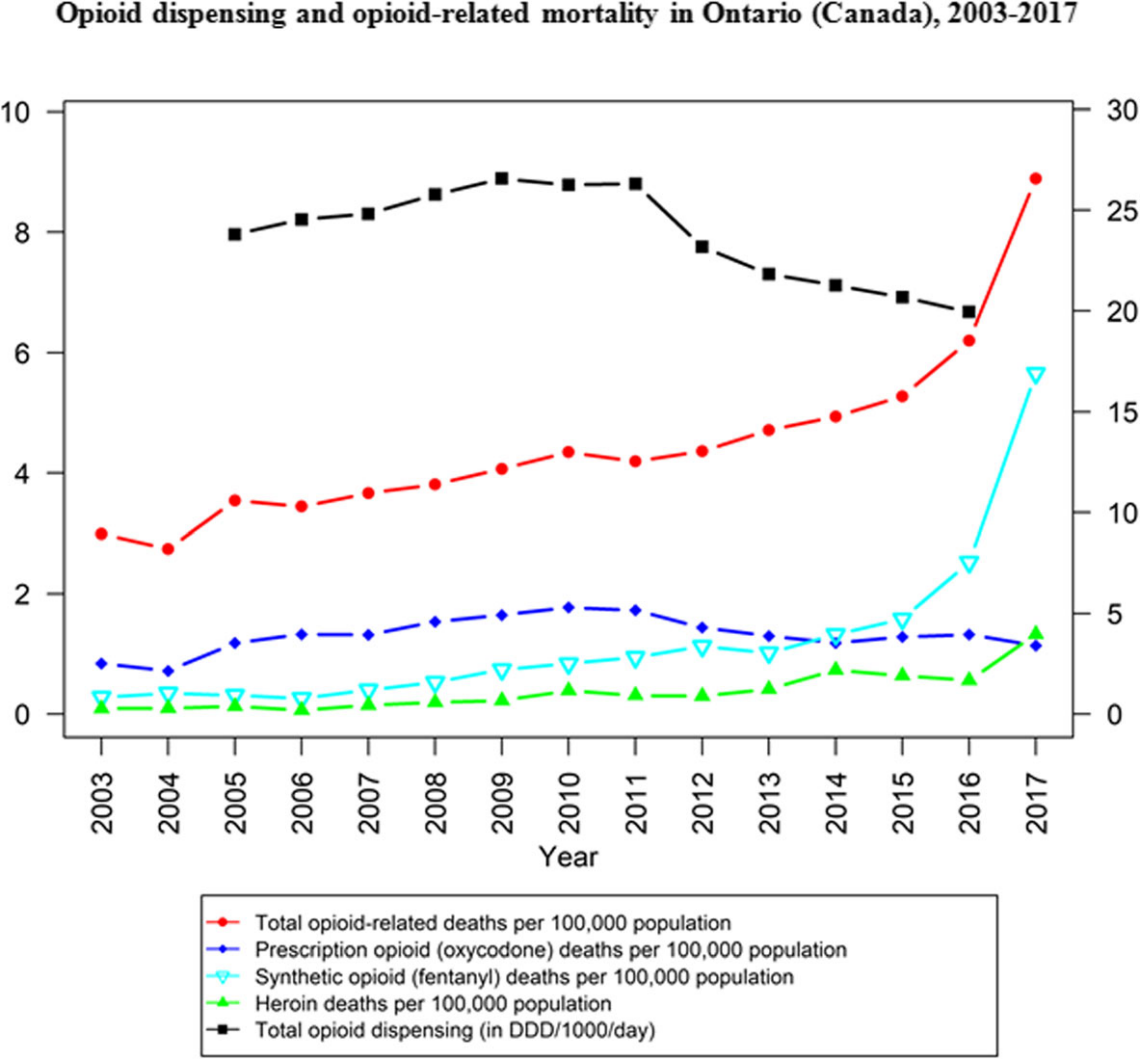
Opioid dispensing and opioid-related mortality in Ontario (Canada), 2003–2017 [40, 68]

**Fig. 3:**
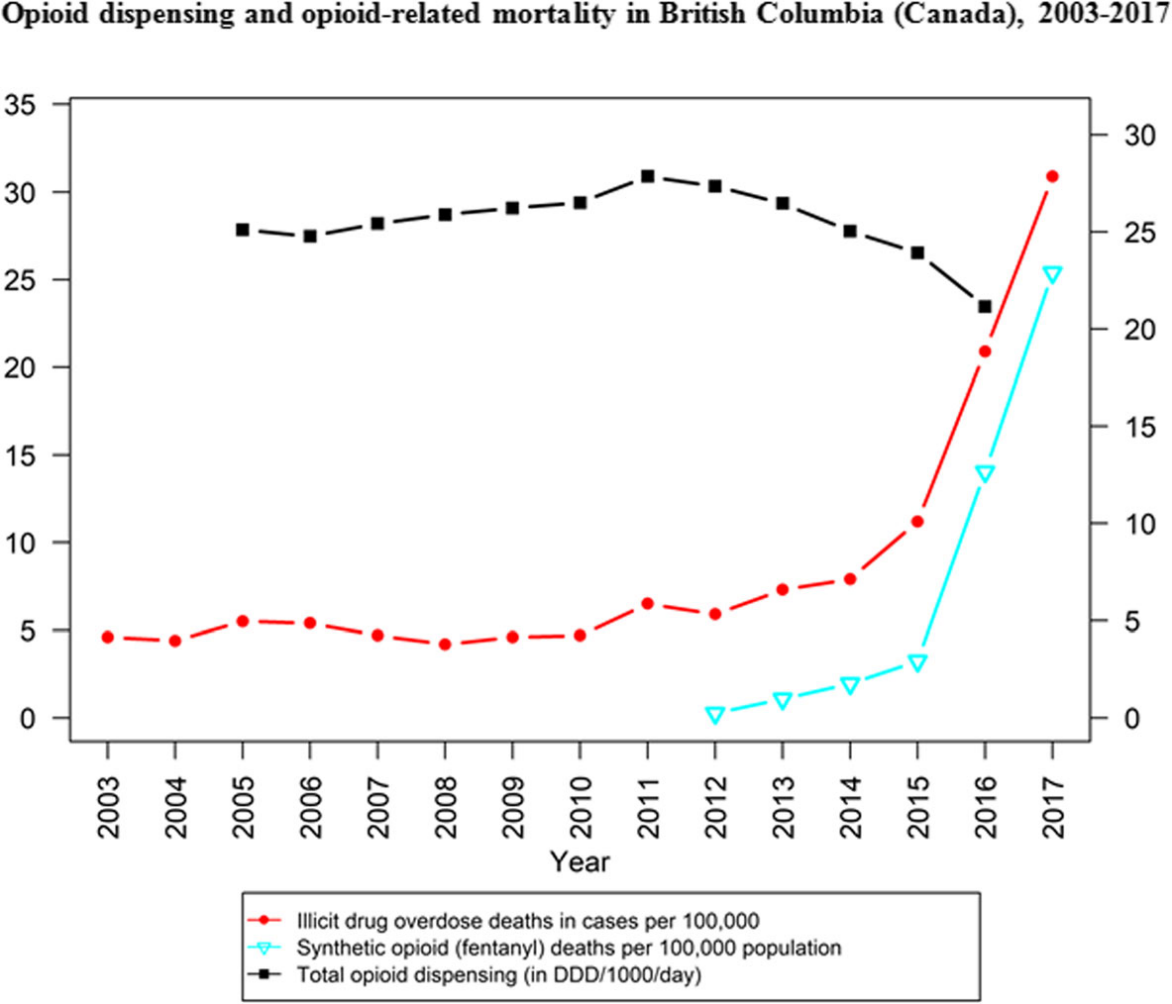
Opioid dispensing and opioid-related mortality in British Columbia (Canada), 2003–2017 [40, 69]

It is around this period of increasing restraints on POs, from about 2012 onwards, when illicit and distinctly potent toxic opioid products (e.g., fentanyl and analogues) initially emerged to make their mark on North American drug markets, with correspondingly rising impact on drug mortality outcomes [48–51]. More likely and plausible than not, this occurred in direct response to the shrinking supplies in non-medical PO products. Despite indications of a resurgence in heroin availability in many (but not all, and mainly focused on those with previously established, viable heroin markets) regions across North America following the implementation of the above described opioid control measures, including evidence that global opium and heroin production (e.g., in Mexico, Asia) appeared to re-expand post-2010, these developments overall were unlikely to sufficiently compensate for the emerging supply gaps to feed existent demand for non-medical opioid use [21, 52, 53]. Multiple epidemiological studies since 2010 have illustrated predominant trajectories of opioid use, mainly from initial non-medical PO to illicit opioid use, commonly involving transitions from non-injecting to injection use, in key sub-populations [54–56]. Among other adverse health risks and outcomes, rising incidences of infectious disease (e.g., Hepatitis C Virus) transmissions have been observed in some settings, especially among younger injection opioid users with a history of non-medical PO use [57–60]. While contracting supplies and the need for more ‘cost-effective’ administration routes more commonly led to opioid use by injection, however, the socio-behavioral profiles of many non-medical opioid users in North America (e.g., involving many non-marginalized users with principally PO-based trajectories) have been such that typical routes of heroin usage (e.g., injection or smoking) presumably were not universally realistic or desirable options [61–65]. Time-trend data for opioid-related mortality in U.S. (Fig. 1) and the Canadian provinces of Ontario (Fig. 2) and British Columbia (Fig. 3) suggest that overdose fatalities related to illicit opioid products began to increase just as medical PO supplies, and related mortality, began to inflect towards decreasing trends across North America. These trend observations lend supporting evidence to the suggestion of possible lateral, substitutive shifts in supply from one opioid category to the other.

There are various case studies and – with some presenting counter-factual scenarios - that provide plausibly supporting evidence for the explanatory observations offered. For example, within Canada, the province of Quebec has traditionally featured substantially lower – by up to a three-fold magnitude - levels of PO dispensing compared to all other provinces, and consequently lower population-level exposure to POs [40, 74, 75]. Notably, Quebec has not seen the dramatic increases in opioid-related mortality, nor in levels of illicit opioid related deaths experienced elsewhere in North America [2]. Similarly, other Anglo-Saxon countries with relatively lower PO consumption levels (e.g. Australia, New Zealand, United Kingdom) have not experienced the extreme spikes in opioid mortality that have been observed in the U.S. and Canada [70, 76–79]. Yet, there remains potentially inconsistent evidence that is difficult to reconcile with the perspectives presented. For example, mortality related to synthetic opioids has barely increased in select U.S. states such as Washington and Oregon - two jurisdictions with traditionally high PO dispensing levels and direct geographical proximity to British Columbia, Canada, an epicentre of synthetic opioid mortality in North America [52]. However, there may indeed exist regionally distinct and separate market dynamics for opioid supply between these jurisdictions despite their geographic proximity.

In the above, we have presented evidence and potential explanatory frameworks to examine the supply side dynamics of the unprecedented opioid mortality epidemic as has been unfolding across North America. A substantial body of data suggests that the current crisis, with excessive numbers of deaths related to synthetic opioids, ought to be understood as an evolutionary consequence of prolonged high PO availability and exposure, followed by substantial restrictions in availability in the context of persistent non-medical opioid use demand at the population level. Likely, these restrictive interventions implemented over the past decade have resulted in growing PO supply gaps in spite of persistent demand, and that were compensated for through an influx of highly potent and toxic illicit opioid products. Notably, while opioid overdose fatality levels have risen steeply due to illicit opioid products in recent years, this does not necessarily imply an expansion in the volume of high-risk opioid use or overdose incidents. Rather, the likelihood of overdose incidents and fatality outcomes has likely become elevated through the use of highly potent and toxic opioid products [8, 21, 48, 52].

Given the relative ease of clandestine chemical production and distribution by the creative and adaptive forces behind illicit drug production and supply, it surely required little ingenuity to effectively recognize and respond to these extensive demands for opioid supply, especially in regions of North America where demand for prescription opioid products for non-medical use had been persistently high for long periods [11, 12, 71, 72]. Relevant analyses of both toxicology and production and supply stream data has suggested that a large proportion of the newly emerging fentanyl and other synthetic opioid products originated mainly in both China and Mexico; from there, facilitated by both relatively simple chemical production processes, small drug quantities involved as well as inadequate monitoring and control of these trafficking activities, either final products or precursors for local synthesizing were clandestinely shipped to and distributed in North America [12, 72, 73, 80]. For exemplary seizure data-based illustration coinciding with above-described illicit opioid supply trends: The amounts of illicit fentanyl products seized at US border entry-points rose from 1 kg in 2013 to 675 kg in 2017; reports of drug enforcement seizures containing fentanyl submitted to state and local laboratories increased from 978 (2013) to 34,000 (2016) [81]. If valid – and rigorous (e.g., epidemiological modelling) analyses ought to further examine these proposed scenarios of the dynamic interplay between different strands of (e.g., prescription and illicit) opioid supply and demand, and the various catastrophic adverse health consequences, in North America – this implies at least two main insights. First, we need to improve our analytical understanding of psychoactive drug markets and their dynamics, especially when both medical and illicit drug products are involved, also because a selective and narrow focus on interventions addressing a single supply source is likely to lead to un-anticipated supply alternatives [82, 83]. This appears especially so when the corresponding ‘demand’ side has not been effectively contained, as has been the case in the present North American crisis scenario. Second, while excessive PO availability had existed in North America for a rather long time, most interventions to curtail these came rather (too) late and/or were insufficient [6]. Certainly, few observers anticipated that PO supplies for non-medical use would be replaced so quickly by an ample availability of exceptionally potent and toxic, illicit opioid products.

## Conclusions

It is becoming increasingly clear that ‘supply side’ considerations are not only essential for our understanding of the present opioid crisis, but also essential for informing effective interventions and solutions delivered on the ground. Concretely, while multiple, extensive ‘demand side’ interventions targeting non-medical opioid use and its adverse health risks (such as naloxone distribution programs, safer consumption facilities, opioid agonist treatment options) have been implemented and expanded, and there is substantial room for further scale-up and select additional intervention programming (e.g., ED-based pharmacotherapy treatment provision or referral [84, 85], their overall impact in reducing opioid-related harms likely has reached natural limits in the persistent high-risk environment dominated by highly toxic, synthetic opioids [7, 8, 52]. Core evidence for this includes persistently high rates of opioid-related mortality across North America [86, 87] combined with socio-behavioral data that, for example, in Canadian locales a majority of opioid-related overdose fatalities occur among individuals using drugs alone where they cannot readily be assisted by emergency measures like naloxone provision [62]. While innovative treatment interventions like injection opioid (heroin)-assisted maintenance therapy – originally initiated in Europe and offered in a few select, small-scale Canadian programs [88–90] – may serve small sub-groups of high-risk patients, scalable, large-scale ‘safer opioid supply’ interventions are required as complementary intervention strategies to mainly behavior- or environment-focused measures for non-medical opioid users at high risk of exposure to illicit, toxic products [8, 52, 91]. Such ‘safer opioid supply’ measures – some of which are proceeding on a local, experimental basis (e.g. in Vancouver) – would provide medical grade opioids products mainly aiming to prevent and replace the use of illicit, toxic opioid products [92, 93]. While this reminds of past considerations on the possible public health benefits of POs replacing illicit opioid use, such ‘emergency measures’ need to be effectively combined with simultaneous comprehensive prevention measures for those many people not currently involved in non-medical opioid use [94].

However, improved empirical understanding of the causal supply dynamics and structures driving the present opioid mortality crisis, and of non-medical drug supply mechanics more generally, are urgently required – if for nothing else than to better prevent such tragedies in the future. An estimated 250,000 lives lost to opioids, and historic reversals in population-level life expectancy for an entire generation of North Americans in barely a decade should provide more than good and sufficient reason for doing so.

## Data Availability

All data used in generating our figures are publicly available and cited in the reference list [40, 66–69].

## Abbreviations

DDD: Defined Daily Dose
PO: Prescription opioid

## References

[1] Center for Disease Control and Prevention. National Center for Health Statistics: Provisional Opioid Overdose Death Counts. 2019 Available from: https://www.cdc.gov/nchs/nvss/vsrr/drug-overdose-data.htm.

[2] Health Canada. Overview of national data on opioid-related harms and deaths. 2018 Available from: https://www.canada.ca/en/health-canada/services/substance-use/problematic-prescription-drug-use/opioids/data-surveillance-research/harms-deaths.html. Retrieved on January 2, 2020.

[3] Case A, Deaton A (2015). Rising morbidity and mortality in midlife among white non-Hispanic Americans in the 21st century. Proc Natl Acad Sci U S A.

[4] Public Health Agency of Canada. The Chief Public Health Officer’s Report on the State of Public Health in Canada 2018: Preventing problematic substance use in youth. Public Health Agency of Canada (PHAC) 2018.

[5] Kolodny A, Courtwright DT, Hwang CS, Kreiner P, Eadie JL, Clark TW (2015). The prescription opioid and heroin crisis: a public health approach to an epidemic of addiction. Annu Rev Public Health.

[6] Fischer B, Rehm J, Tyndall M (2016). Effective Canadian policy to reduce harms from prescription opioids: learning from past failures. CMAJ..

[7] Wood E (2018). Strategies for reducing opioid-overdose deaths - lessons from Canada. N Engl J Med.

[8] Frank RG, Pollack HA (2017). Addressing the fentanyl threat to public health. N Engl J Med.

[9] Lucyk SN, Nelson LS (2017). Novel synthetic opioids: an opioid epidemic within an opioid epidemic. Ann Emerg Med.

[10] Fischer B, Vojtila L, Rehm J (2018). The 'fentanyl epidemic' in Canada - some cautionary observations focusing on opioid-related mortality. Prev Med.

[11] Armenian P, Vo KT, Barr-Walker J, Lynch KL. Fentanyl, fentanyl analogs and novel synthetic opioids: A comprehensive review. Neuropharmacology. 2018;134(Pt A):121–32. 10.1016/j.neuropharm.2017.10.016 29042317

[12] Suzuki J, El-Haddad S (2017). A review: fentanyl and non-pharmaceutical fentanyls. Drug Alcohol Depend.

[13] BC Centre for Disease Control. Opioid Overdose Fact Sheet. 2018. Available from: http://www.bccdc.ca/health-professionals/data-reports/overdose-response-reports. Retrieved: January 2, 2020.

[14] Alberta Health Services. Opioids and Substances of Misuse - Alberta Report, 2017 Q4. 2018.

[15] Hedegaard H, Minino AM, Warner M. Drug Overdose Deaths in the United States, 1999-2017. NCHS Data Brief 2018(329):1–8. 30500323

[16] National Institute on Drug Abuse. National Drug Overdose Deaths Involving Cocaine, by Opioid Involvement – Number Among All Ages, 1999–2017. National Institute on Drug Abuse (NIDA);2019. Available from: https://www.drugabuse.gov/related-topics/trends-statistics/overdose-death-rates. Retreived on January 3, 2020.

[17] Duran EZ, B.C. marks 2017 as deadliest O.D. death year in provincial history. Global News. 2018.

[18] Caulkins JP (1998). Reuter P. What Price Data Tell Us about Drug Markets.

[19] Degenhardt L, Reuter P, Collins L, Hall W (2005). Evaluating explanations of the Australian 'heroin shortage'. Addiction..

[20] Weatherburn D, Jones C, Freeman K, Makkai T (2003). Supply control and harm reduction: lessons from the Australian heroin 'drought'. Addiction..

[21] Ciccarone D. Fentanyl in the US heroin supply: a rapidly changing risk environment. International Journal of Drug Policy. 2017;46:107–11. 10.1016/j.drugpo.2017.06.010 PMC5742018 28735776

[22] Mars SG, Rosenblum D, Ciccarone D. Illicit fentanyls in the opioid street market: desired or imposed? Addiction. 2018;114(5):774–78. 10.1111/add.14474 PMC6548693 30512204

[23] Fischer B, Argento E. Prescription opioid related misuse, harms, diversion and interventions in Canada: a review. Pain Physician. 2012;15(3 Suppl):Es191–203. 22786457

[24] International Narcotics Control Board (2015). Availability of internationally controlled drugs: ensuring adequate access for medical and scientific purposes - chapter II: narcotic drugs - availability of opioid analgesics.

[25] Han B, Compton WM, Jones CM, Cai R (2015). Nonmedical prescription opioid use and use disorders among adults aged 18 through 64 years in the United States, 2003-2013. JAMA..

[26] Nosyk B, Marshall BD, Fischer B, Montaner JS, Wood E, Kerr T (2012). Increases in the availability of prescribed opioids in a Canadian setting. Drug Alcohol Depend.

[27] Fischer B, Rehm J, Patra J, Cruz MF (2006). Changes in illicit opioid use across Canada. CMAJ..

[28] Paulozzi LJ, Budnitz DS, Xi Y (2006). Increasing deaths from opioid analgesics in the United States. Pharmacoepidemiol Drug Saf.

[29] Jones CM, Mack KA, Paulozzi LJ (2013). Pharmaceutical overdose deaths, United States, 2010. JAMA..

[30] Dhalla IA, Mamdani MM, Sivilotti ML, Kopp A, Qureshi O, Juurlink DN (2009). Prescribing of opioid analgesics and related mortality before and after the introduction of long-acting oxycodone. CMAJ..

[31] Fischer B, Jones W, Rehm J (2013). High correlations between levels of consumption and mortality related to strong prescription opioid analgesics in British Columbia and Ontario, 2005-2009. Pharmacoepidemiol Drug Saf.

[32] Paulozzi LJ, Mack KA, Hockenberry JM (2014). Vital signs: variation among states in prescribing of opioid pain relievers and benzodiazepines - United States, 2012. MMWR Morb Mortal Wkly Rep.

[33] Manchikanti L, Helm S, 2nd, Fellows B, Janata JW, Pampati V, Grider JS, et al. Opioid epidemic in the United States. Pain Physician. 2012;15(3 Suppl):Es9–38. 22786464

[34] Volkow ND, McLellan AT (2016). Opioid abuse in chronic pain--misconceptions and mitigation strategies. N Engl J Med.

[35] Fischer B, Gooch J, Goldman B, Kurdyak P (2014). Rehm. Non-medical prescription opioid use, prescription opioid-related harms and public health in Canada: an update 5 years later. Canadian Journal of Public Health.

[36] Reifler LM, Droz D, Bailey JE, Schnoll SH, Fant R, Dart RC (2012). Do prescription monitoring programs impact state trends in opioid abuse/misuse?. Pain Med.

[37] Franklin GM, Mai J, Turner J, Sullivan M, Wickizer T, Fulton-Kehoe D (2012). Bending the prescription opioid dosing and mortality curves: impact of the Washington state opioid dosing guideline. Am J Ind Med.

[38] Surratt HL, O'Grady C, Kurtz SP, Stivers Y, Cicero TJ, Dart RC (2014). Reductions in prescription opioid diversion following recent legislative interventions in Florida. Pharmacoepidemiol Drug Saf.

[39] Guy GP, Zhang K, Bohm MK, Losby J, Lewis B, Young R (2017). Vital signs: changes in opioid prescribing in the United States, 2006-2015. MMWR Morb Mortal Wkly Rep.

[40] Fischer B, Jones W, Vojtila L, Kurdyak P (2018). Patterns, changes, and trends in prescription opioid dispensing in Canada, 2005-2016. Pain Physician.

[41] Fischer B, Jones W, Rehm J (2014). Trends and changes in prescription opioid analgesic dispensing in Canada 2005-2012: an update with a focus on recent interventions. BMC Health Serv Res.

[42] Schieber L, Guy G, Seth P, Young R, Mattson C, Milkosz C, et al. Trends and Patterns of Geographic Variation in Opioid Prescribing Practices by State, United States, 2006-2017. JAMA Network Open. 2019:2(3):e190665. 10.1001/jamanetworkopen.2019.0665 PMC6484643 30874783

[43] Surratt H, Kurtz S, Cicero T, Dart R, Baker G, Vorsanger G (2013). Street prices of prescription opioids diverted to the illicit market: data from a national surveillance program. J Pain.

[44] Severtson SG, Bartelson BB, Davis JM, Munoz A, Schneider MF, Chilcoat H, et al. Reduced abuse, therapeutic errors, and diversion following reformulation of extended-release oxycodone in 2010. The Journal of Pain. 2013;14(10):1122–30. 10.1016/j.jpain.2013.04.011 23816949

[45] Fischer B. Prescription opioid use, harms and interventions in Canada: a review update of new developments and findings since 2010. 2015;18:E605-E14. 26218951

[46] Fischer B, Varatharajan T, Shield K, Rehm J, Jones WJ (2018). Crude estimates of prescription opioid-related misuse and use disorder populations towards informing intervention system need in Canada. Drug Alcohol Depend.

[47] Saloner B, Karthikeyan SJJ. Changes in substance abuse treatment use among individuals with opioid use disorders in the United States, 2004–2013. JAMA. 2015;314(14):1515–7. 10.1001/jama.2015.10345 26462001

[48] O’Donnell JK, Gladden RM, Seth PJ. Trends in deaths involving heroin and synthetic opioids excluding methadone, and law enforcement drug product reports, by census region—United States, 2006–2015. Morbidity and Mortality Weekly Report. 2017;66(34):897. 10.15585/mmwr.mm6634a2 PMC5657786 28859052

[49] Gladden R. Fentanyl law enforcement submissions and increases in synthetic opioid–involved overdose deaths—27 states, 2013–2014. MMWR. 2016;65. 10.15585/mmwr.mm6533a2 27560775

[50] Gomes T, Khuu W, Martins D, Tadrous M, Mamdani MM, Paterson JM (2018). Contributions of prescribed and non-prescribed opioids to opioid related deaths: population based cohort study in Ontario. Canada..

[51] BC Coroners Service. Fentanyl-Detected Illicit Drug Overdose Deaths: January 1, 2012 to September 30, 2018. British Columbia. 2018.

[52] Fairbairn N, Coffin PO, Walley M (2017). Naloxone for heroin, prescription opioid, and illicitly made fentanyl overdoses: challenges and innovations responding to a dynamic epidemic. Int J Drug Policy.

[53] Dart RC, Surratt HL, Cicero TJ, Parrino MW, Severtson SG. Bucher-Bartelson B, et al. Trends in opioid analgesic abuse and mortality in the United States. NEJM. 2015;372(3):241–8. 10.1056/NEJMsa1406143 25587948

[54] Compton WM, Jones CM, Baldwin J (2016). Relationship between nonmedical prescription-opioid use and heroin use. N Engl J Med.

[55] Mars SG, Bourgois P, Karandinos G, Montero F, Ciccarone D. “Every ‘never’I ever said came true”: transitions from opioid pills to heroin injecting. Int J Drug Policy. 2014;25(2):257–66. 10.1016/j.drugpo.2013.10.004 PMC3961517 24238956

[56] DeBeck K, Wood E, Dong H, Dobrer S, Hayashi K, Montaner J (2016). Non-medical prescription opioid use predicts injection initiation among street-involved youth. Internaional Journal of Drug Policy.

[57] Suryaprasad AG, White JZ, Xu F, Eichler B-A, Hamilton J, Patel A (2014). Emerging epidemic of hepatitis C virus infections among young nonurban persons who inject drugs in the United States, 2006–2012. J Clinical Infectious Diseases.

[58] Hadland SE, DeBeck K, Kerr T, Feng C, Montaner JS, Wood E (2014). Prescription opioid injection and risk of hepatitis C in relation to traditional drugs of misuse in a prospective cohort of street youth. BMJ Open.

[59] Bruneau J, Roy É, Arruda N, Zang G, Jutras-Aswad D (2012). The rising prevalence of prescription opioid injection and its association with hepatitis C incidence among street-drug users. Addiction..

[60] Havens JR, Lofwall MR, Frost SD, Oser CB, Leukefeld CG, Crosby RA (2013). Individual and network factors associated with prevalent hepatitis C infection among rural Appalachian injection drug users. Am J Public Health.

[61] Peppin J, Coleman J. Characterization of prescription opioid abuse in the United States: focus on route of administration. Journal of Pain & Palliative Care Pharmacotherapy. 2012;26(4):348–61. 10.3109/15360288.2012.734905 23675595

[62] BC Coroners Service. llicit Drug Overdose Deaths in B.C.: Findings of Coroners' Investigations. Vancouver: BC Coroners Service; 2018.

[63] Monico LB, Mitchell SG. Patient perspectives of transitioning from prescription opioids to heroin and the role of route of administration. Substance abuse treatment, prevention, policy. 2018;13(1):4. 10.1186/s13011-017-0137-y PMC5789586 29378623

[64] Harocopos A, Allen B, Paone D (2016). Circumstances and contexts of heroin initiation following non-medical opioid analgesic use in New York City. Int J Drug Policy.

[65] Jones CM, Christensen A, Gladden RM (2017). Increases in prescription opioid injection abuse among treatment admissions in the United States, 2004–2013. Drug Alcohol Depend.

[66] Centers for Disease Control and Prevention. U.S. Prescribing Rate Maps. 2018. https://www.cdc.gov/drugoverdose/ maps/rxrate-maps.html. Accessed 24 Feb 2019.

[67] National Institute on Drug Abuse. Overdose Death Rates in 2018. 2018. https://www.drugabuse.gov/related-topics/trends-statistics/ overdose-death-rates. Accessed 24 Feb 2019.

[68] Public Health Ontario. Rates of opioid-related mortality, Ontario, Canada, 2003–2017. British Columbia. 2018. https://www.publichealthontario.ca/en/data-and-analysis/substance-use/interactive-opioid-tool. Accessed 24 Feb 2019.

[69] British Columbia Coroners Service. Illicit drug overdose deaths and death rate per 100,000 population. 2019. https://www2.gov.bc.ca/assets/gov/birth-adoption-death-marriage-and-divorce/deaths/coroners-service/statistical/illicit-drug.pdf. Accessed 24 Feb 2019.

[70] McKeown HE, Rook TJ, Pearson JR, Jones OAH (2018). Is Australia ready for fentanyl?. Science & justice: Journal of the Forensic Science Society.

[71] Schueler HE (2017). Emerging synthetic fentanyl analogs. Academic Forensic Pathology.

[72] Beletsky L, Davis CS (2017). Today's fentanyl crisis: Prohibition's Iron law, revisited. The International journal of drug policy.

[73] Armenian P, Vo KT, Barr-Walker J, Lynch KL (2018). Fentanyl, fentanyl analogs and novel synthetic opioids: a comprehensive review. Neuropharmacology..

[74] Fischer B, Jones W, Krahn M, Rehm J (2011). Differences and over-time changes in levels of prescription opioid analgesic dispensing from retail pharmacies in Canada, 2005–2010. Pharmacoepidemiol Drug Saf.

[75] Canadian Institute for Health Information. Pan-Canadian Trends in the Prescribing of Opioids, 2012 to 2016. Ottawa, ON: Canadian Institute for Health Information (CIHI); 2017.

[76] Blanch B, Pearson SA, Haber P (2014). An overview of the patterns of prescription opioid use, costs and related harms in Australia. Br J Clin Pharmacol.

[77] Weisberg DF, Becker WC, Fiellin DA (2014). Stannard. Prescription opioid misuse in the United States and the United Kingdom: cautionary lessons. Int J Drug Policy.

[78] Mounteney J, Giraudon I, Denissov G (2015). Griffiths. Fentanyls: are we missing the signs? Highly potent and on the rise in Europe. Int J Drug Policy.

[79] van Amsterdam J, van den Brink W (2015). The misuse of prescription opioids: a threat for Europe?. Current Drug Abuse Reviews.

[80] Garza A. Illicitly produced fentanyl: a growing cause of synthetic opioid deaths: pharmacy times; 2018. Available from: https://www.pharmacytimes.com/publications/issue/2018/august2018/illegally-produced-fentanyl-a-growing-cause-of-synthetic-opioid-deaths. Accessed: January 2, 2020.

[81] RAND (2018). Evolution of the U.S.

[82] Babor T, Caulkins J, Fischer B, Foxcroft D, Humphreys K, Medina-Mora ME, et al. Drug policy and the public good: Oxford University Press; 2018.

[83] Alpert A, Powell D, Pacula RL (2018). Supply-side drug policy in the presence of substitutes: evidence from the introduction of abuse-deterrent opioids. Am Econ J Econ Pol.

[84] D’Onofrio G, Chawarski MC, O’Connor PG, Pantalon MV, Busch SH, Owens PH, et al. Emergency department-initiated buprenorphine for opioid dependence with continuation in primary care: outcomes during and after intervention. J Gen Intern Med 2017;32(6):660–6. 10.1007/s11606-017-3993-2 PMC5442013 28194688

[85] Samuels EA, Dwyer K, Mello MJ, Baird J, Kellogg AR, Bernstein E (2016). Emergency department-based opioid harm reduction: moving physicians from willing to doing. Acad Emerg Med.

[86] Scholl L, Seth P, Kariisa M, Wilson N, Baldwin G (2019). Drug and opioid-involved overdose deaths - United States, 2013–2017.

[87] Government of Canada. Opioid-related harms in Canada Ottawa, ON: Government of Canada; 2019. Available from: https://infobase.phac-aspc.gc.ca/datalab/national-surveillance-opioid-mortality.html. Accessed: January 2, 2020.

[88] Strang J, Groshkova T, Uchtenhagen A, van den Brink W, Haasen C, Schechter MT (2015). Heroin on trial: systematic review and meta-analysis of randomised trials of diamorphine-prescribing as treatment for refractory heroin addiction. Br J Psychiatry.

[89] Oviedo-Joekes E, Brissette S, Marsh DC, Lauzon P, Guh D, Anis A (2009). Diacetylmorphine versus methadone for the treatment of opioid addiction. N Engl J Med.

[90] Ferri M, Davoli M, Perucci CA. Heroin maintenance for chronic heroin-dependent individuals. J Cochrane Database of Systematic Reviews. 2010;8. 10.1002/14651858.CD003410.pub3 20687073

[91] Fischer B, Pang M, Tyndall M. Applying principles of injury and infectious disease control to the opioid mortality epidemic in North America: critical intervention gaps. J Public Health. 2019:fdz162. 10.1093/pubmed/fdz162 PMC7685850 31822889

[92] Tyndall M (2018). An emergency response to the opioid overdose crisis in Canada: a regulated opioid distribution program. CMAJ..

[93] Fischer B, Pang M, Tyndall M (2019). The opioid death crisis in Canada: crucial lessons for public health. Lancet Public Health.

[94] Fischer B, Gittins J, Kendall P, Rehm J (2009). Thinking the unthinkable: could the increasing misuse of prescription opioids among street drug users offer benefits for public health?. Public Health.

